# In vitro study of the potential protection of sound enamel against demineralization

**DOI:** 10.1186/s40510-015-0080-2

**Published:** 2015-05-22

**Authors:** Mona A Montasser, Noha A El-Wassefy, Mahasen Taha

**Affiliations:** Orthodontic Department, Faculty of Dentistry 35516, Mansoura University, Mansoura, Egypt; Dental Biomaterials Department, Faculty of Dentistry 35516, Mansoura University, Mansoura, Egypt

**Keywords:** Enamel, Demineralization, Protective methods

## Abstract

**Background:**

The objective of this study was to study the potential protection effect of different treatments against sound enamel demineralization around orthodontic brackets.

**Methods:**

This is an in vitro randomized controlled study; artificial enamel demineralization of human premolars was created and compared with reference to control. The three materials used for enamel treatment were resin infiltrate (ICON), fluoridated varnish (Clinpro), and the self-etch primer system (Transbond Plus Self-Etch Primer). Fifty premolars divided equally into five groups were included in the study for quantitative surface micro-hardness assessment using a micro-hardness tester (MHT). Qualitative assessment of the enamel demineralization with a polarized light microscope (PLM) was also used. Enamel was demineralized by subjecting the specimens to cycling between artificial saliva solution and a demineralizing solution for 21 days.

**Results:**

The mean Vickers hardness in kgf/mm^2^ was as follows: intact enamel = 352.5 ± 13.8, demineralized enamel = 301.6 ± 34.0, enamel treated with Clinpro = 333.6 ± 18.0, enamel treated with SEP = 370.7 ± 38.8, and enamel treated with ICON = 380.5 ± 53.8.

**Conclusions:**

ICON, Clinpro, and Transbond Plus Self-Etch Primer (TPSEP) increased enamel resistance to demineralization. Attempting to protect the enamel around the orthodontic brackets could be done by applying a preventive material before bonding, if not compromising the bond strength, the orthodontic brackets.

## Background

Demineralization and appearance of white spot lesions around brackets and bands have become a major concern during orthodontic treatment especially with the potential of these lesions to develop into caries when oral hygiene maintenance is compromised. During orthodontic treatment, an acidic environment develops at the periphery of the orthodontic brackets and bands due to accumulation of bacterial plaque. Enamel demineralization has been repeatedly reported [1–3], and efforts continue to develop ways to prevent development of white spot lesions that are not only unaesthetic but also remineralize poorly increasing the risk of developing carious lesions [4, 5]. Increasing the resistance of the enamel in these areas would control development of white spot lesions.

Multiple preventive agents had been tested over years to evaluate their effectiveness in prevention and treatment of white spot lesions associated with orthodontic treatment [6–11]. It was assumed that the most efficient method of delivering preventive agents during orthodontic treatment would be one independent of patient compliance and specific to those areas most susceptible to demineralization [12]. These included adhesives and cements containing fluoride (F), casein phosphopeptide-amorphous calcium phosphate (CPP-ACP), or amorphous calcium phosphate (ACP); fluoride applications; and sealants. Fluoride has been proven to be effective in fighting demineralization [13, 14] that Rølla et al. [13] considered it the only factor that explains the caries reduction in recent years with a synergistic effect from the improved oral hygiene. Fluoride has been found to be effective in reducing the development of white spot lesions associated with fixed orthodontic treatment [15, 16]. Therefore, incorporating preventive agents in orthodontic bonding composite was considered a potential method to reduce white spot lesions during orthodontic treatment even with the general agreement that it is not simple to predict what might occur when the bonding adhesive is used in the demanding environment of the oral cavity [17–19]. Clinpro, a fluoridated varnish, has been introduced to the market and supposed to be most beneficial in a neutral pH environment. Sealants were suggested as protective enamel agents that do not require patient cooperation, but sealants on areas adjacent to brackets are subject to physical challenges as tooth brushing and acid attacks that limit their effect [20, 21]. Recently, resin infiltrants were found to decrease the dissolution of enamel and so limit the appearance of white spot lesions. In an in vitro study to compare a conventional adhesive, a caries infiltrant (ICON), and a combination of both in resisting demineralization, it was found that in both sound enamel and artificial caries lesions, the application of the caries infiltrant was effective in protecting the enamel against dissolution [22].

Systematic reviews of previous studies found a lack of reliable evidence on a protocol or a method to protect the enamel against development of white spot lesions during orthodontic treatment or to remineralize post-orthodontic white spot lesions [23, 24]. The objective of this study was to study the potential protection of different treatments against sound enamel demineralization around orthodontic brackets.

## Methods

This is an in vitro randomized controlled study; artificial enamel demineralization of human premolars was created and compared with reference to control. Quantitative surface micro-hardness assessment of the specimens was done with a digital display Vickers micro-hardness tester (MHT) (Model HVS-50, Laizhou Huayin Testing Instrument Co., Ltd., China). The study also used a qualitative polarized light microscope (PLM) (Olympus dual stage polarized light microscope, Model BH-2, Dualmont Corporation, Minneapolis, MN).

The materials used in this study together with the study design are given in Table 1. The three materials used for enamel treatment were Clinpro (3M Unitek, Monrovia, CA, USA), a fluoridated varnish containing 5 % sodium fluoride; ICON (DMG, Hamburg, Germany), a resin infiltrant; and Transbond Plus Self-Etch Primer (TPSEP) (3M Unitek, Monrovia, CA, USA). All materials were used according to the manufacturers’ instructions.

**Table 1:** Study groups and examination methods

Group	Description	Treatment duration	Examination method	
1	Enamel, no demineralization, no treatment (control)	21 days	MHT	PLM
2	Enamel, demineralization, no treatment (control)			
3	Enamel, demineralization, ICON treatment			
4	Enamel, demineralization, Clinpro treatment			
5	Enamel, demineralization, SEP treatment			

The software EpiCalc 2000 version 1.02 (Brixton Books, Brixton, UK) indicated 10 specimens for each group to be a reliable sample size at power 80 % and confidence interval 95 %. For micro-hardness testing, 50 specimens were prepared and then randomly assigned to 5 groups of 10. A similar sample was prepared for the PLM part of the study. Specimens’ preparation included separation of the crown from the root, removing any calculus or debris, and polishing with non-fluoride prophylaxis. The crown was then sectioned mesio-distally with a diamond separating disc leaving only a thin layer of the underlying dentin. For PLM examination, 140- to 160-μm-thick sections were prepared from each tooth segment.

To create artificial carious lesions of the enamel, artificial saliva solution [19] was prepared consisting of 20 mmol/L NaHCO_3_, 3 mmol/L NaH_2_PO_4_, and 1 mmol/L CaCl_2_ at neutral pH. Alongside, a demineralizing solution consisting of 2.2 mmol/L Ca^2+^, 2.2 mmol/L PO4^3−^, and 50 mmol/L acetic acid at pH 4.4 was prepared. Deionized water was used in the preparation of the two solutions. Solutions were measured using a pH/mV meter (Accumet Portable, Fisher Scientific, Pittsburgh, PA) and calcium electrode (Thermo Electron Co., Beverly, MA). The specimens were placed in the prepared artificial saliva solution for 12 h before subjecting them to the demineralizing solution. The specimens were subjected to cycling between the artificial saliva and the demineralizing solutions for 21 days [25].

Surface micro-hardness of the specimens was determined using MHT with a Vickers diamond indenter and a ×20 objective lens. A load of 200 g was applied to the surface of the specimens for 10 s. Three indentations equally placed over a circle and each no closer than 0.5 mm to the adjacent indentations were made on the surface of each specimen. The diagonals’ length of the indentations was measured by a built-in scaled microscope, and Vickers values were converted into micro-hardness values. Micro-hardness was obtained using the following equation: HV = 1.854 *P*/*d*^2^, where HV is Vickers hardness in kgf/mm^2^, *P* is the load in kgf, and *d* is the length of the diagonals in mm. For the qualitative part of the current study, the prepared specimens were analyzed with the PLM. Each specimen was wetted with deionized water and then was oriented longitudinally on a glass cover slide and mounted, and the stage rotated to allow maximum illumination. The area of demineralization was centered in the field of view and photographed under maximum illumination at ×10 magnification.

Statistical analysis of the results of micro-hardness testing included one-way ANOVA followed by least significant difference (LSD) post-hoc analysis for comparisons between groups.

## Results

**Fig. 1:**
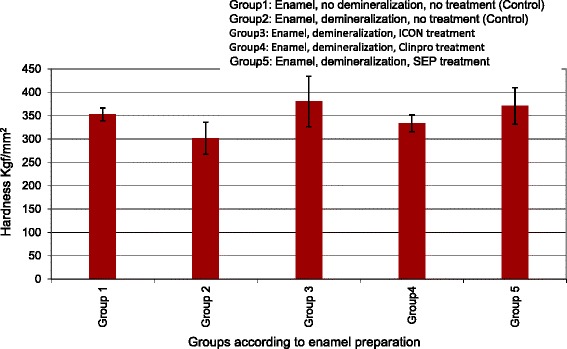
Mean ± SD of the enamel hardness (kgf/mm^2^) of the five tested groups

Descriptive statistics of the enamel hardness of each group are presented in Table 2 and Fig. 1. The demineralized untreated enamel group followed by the Clinpro group showed the lowest hardness values of the enamel surface. The ICON group, the SEP group, and the intact undemineralized enamel group showed the highest hardness values of the enamel surface in descending order.

**Table 2:** Descriptive statistics of the enamel hardness

Group	*N*	Mean (kgf/mm^2^)	SD	Minimum	Maximum
1	10	352.5	±13.8	334.8	370.0
2	10	301.6	±34.0	227.3	341.3
3	10	380.5	±53.8	309.4	679.4
4	10	333.6	±18.0	314.3	373.4
5	10	370.7	±38.8	304.1	660.1

*F* = 3.42; *P* = 0.009. Significance at *P* ≤ 0.05

The one-way ANOVA (Table 2) indicated a significant difference in the enamel hardness between groups (*P* = 0.009). There was no significant difference between the intact enamel and the demineralized enamel groups (*P* = 0.09), the Clinpro group (*P* = 0.52), the SEP group (*P* = 0.19), and the ICON group (*P* = 0.10). However, there was a significant difference between the demineralized enamel group and both the SEP group (*P* = 0.004) and the ICON group (*P* = 0.001).

On the other hand, there was no significant difference between the two treated groups: Clinpro group and SEP group (*P* = 0.05), and Clinpro group and ICON group (*P* = 0.24). The SEP group and ICON group were not significantly different (*P* = 0.71).

**Fig. 2:**
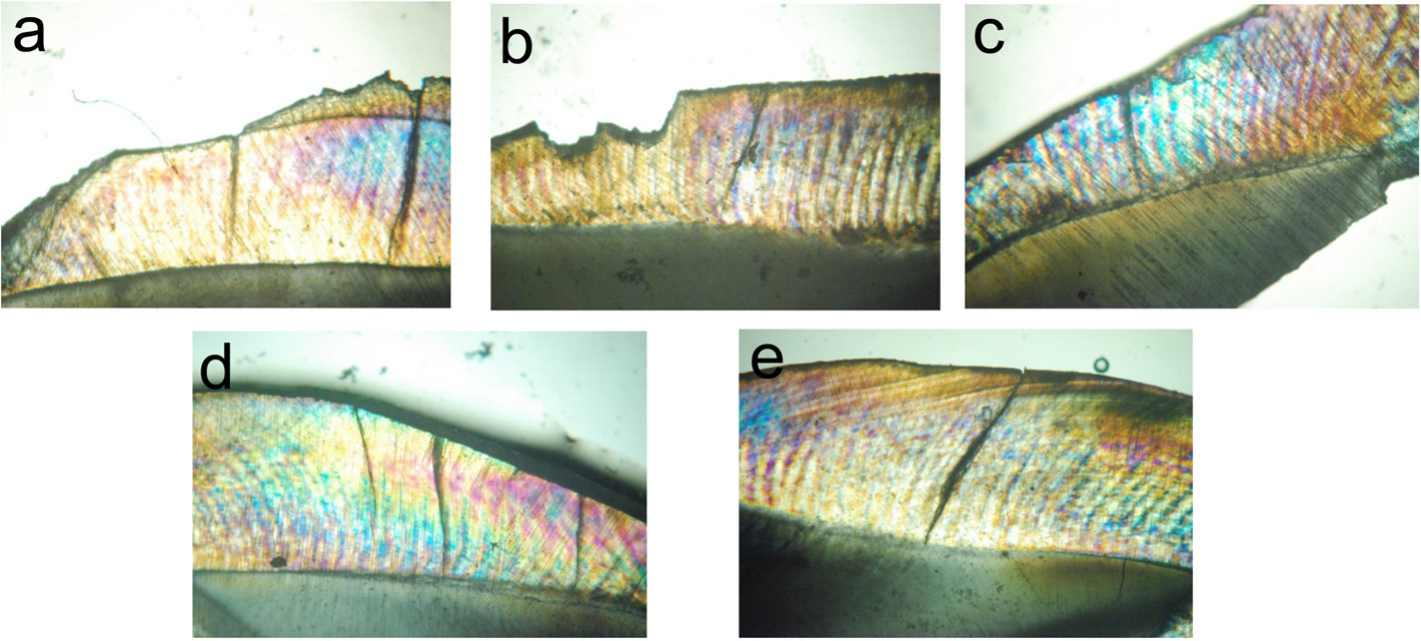
Showing polarized light microscope micrograph at ×10 magnification. **a** Intact enamel. **b** Demineralized enamel. **c** Enamel treated with ICON. **d** Enamel treated with Clinpro. **e** Enamel treated with SEP

A representative photomicrograph for each of the five groups from polarized light microscopy is shown in Fig. 2. The photomicrographs showed that all the three groups of ICON, Clinpro, and SEP were less affected by the demineralization, with smaller lesions and a bright color.

## Discussion

Micro-hardness is a linear function of the local calcium content [26] that can be used not only as a comparative measure of hardness but also as a direct measure of mineral gain or loss as a consequence of demineralization and remineralization [27, 28]. The method was proved as valid and useful for assessment of the changes in enamel surface demineralization [29, 30].

In the current study, the mean enamel hardness of the control group was 352.5 ± 13.8 kgf/mm^2^, while that of the demineralized enamel was 301 ± 34.0 Kgf/mm^2^. Applying the fluoridated varnish Clinpro to the enamel surface before demineralization helped to preserve the enamel hardness to some extent as the enamel hardness in this group remained less than that in the control group; the mean enamel hardness in this group was 333.6 ± 18.0 kgf/mm^2^. Clinpro™ is a white varnish with tri-calcium phosphate (TCP) ingredient to deliver fluoride, phosphate, and calcium. According to the manufacturer, a protective barrier is created around this ingredient during manufacturing, and as the varnish flows on the teeth, it comes into contact with saliva, breaking down the protective barrier and so makes calcium, phosphate, and fluoride ions available to the teeth to decrease the demineralization. Although enamel treated with Clinpro™ was not as hard as the intact untreated enamel, the change might be clinically significant taking into consideration the long duration of orthodontic treatment that on average ranges from 2 to 3 years. Multiple applications of Clinpro over the period of orthodontic treatment may be recommended for further enhancing its protective effect.

The increased surface hardness of the enamel in the ICON group (380.5 ± 53.8 kgf/mm^2^) could be interpreted in light of the mode of action of this material. The low-viscosity light-cured resin material infiltrates the etched enamel surface creating a barrier on the enamel surface, and it is this superficial layer that increases the enamel surface hardness and subsequently increases the resistance to surface demineralization and the development of white spot lesions. Earlier studies as the study of Yetkiner et al. [31] found that the use of low-viscosity caries infiltrant ICON increased sound enamel resistance to demineralization. The mean enamel hardness in group 5 in which the specimens were treated with TPSEP and then subjected to the same protocol of demineralization used in the other groups was 370.7 ± 38.8 kgf/mm^2^. This group, therefore, presented the second highest mean enamel hardness in the current study. TPSEP is a F-releasing self-etch primer, and it acts as other self-etch primers through three mechanisms to stop the etching process and complete the priming: (1) the acid groups attached to the etching monomers are neutralized by forming a complex with the calcium from the hydroxyapatite; (2) the viscosity increases with the air drying, slowing the transport of acid groups to the enamel interface; and (3) the primer polymerizes and the transport of acid groups to the interface stops [32]. The results of the current study suggest that it is not only the effect of the F that increased the enamel hardness but the polymerized surface layer also contributed to this effect. Both group 3 (ICON) and group 5 (SEP) showed increased enamel hardness compared to group 2 (demineralized enamel) and even to group 1 (untreated enamel); this could be contributed by the polymerized surface layer.

On the other hand, the polarized light microscope, with its unique ability to deliver information about the submicroscopic structure of the material under examination utilizing polarized light to form highly magnified images, could qualitatively show the areas of mineral loss and mineral gain represented by areas with different porosities and birefringence [33, 34]. The results of the PLM photomicrographs, as shown in Fig. 2, supported the micro-hardness results; the three groups of ICON, Clinpro, and SEP showed better resistance to enamel demineralization. The photomicrographs showed smaller lesions in the three groups where the enamel was treated with ICON, Clinpro, and SEP compared to the demineralized untreated enamel.

Attempting to protect the enamel around orthodontic brackets could be done through different techniques that each could be effective. This could be by applying a preventive material before bonding the orthodontic brackets or around the brackets after bonding. Regarding the three materials used in the current study, the use of self-etch primers for orthodontic bracket bonding is well documented and has proven not to compromise bond strength [35–37]. A previous study which tested the effect of using ICON and Clinpro before bonding orthodontic brackets with self-etch primer and conventional adhesive systems on the shear bond strength found no significant effect [38].

However, although the observed increase in the enamel hardness would logically suggest more resistance to demineralization, it is not possible to simply expect a solution to the problem of developing white spot lesions during orthodontic treatment with the use of these materials. A systematic review of in vivo studies on the caries-inhibiting effect of preventive measures during orthodontic treatment with fixed appliances considered the effect significant if only it was over 50 % [24].

## Conclusions

ICON, Clinpro, and TPSEP increased enamel resistance to demineralization. Attempting to protect the enamel around the orthodontic brackets could be done by applying a preventive material before bonding, if not compromising the bond strength, the orthodontic brackets.
